# Ferroptosis in thyroid cancer: mechanisms, current status, and treatment

**DOI:** 10.3389/fonc.2025.1495617

**Published:** 2025-01-23

**Authors:** Wenzhi Tian, Xi Su, Chenchen Hu, Dong Chen, Peng Li

**Affiliations:** ^1^ Department of Thyroid and Breast Surgery, Peking University Shenzhen Hospital, Peking University-The Hong Kong University of Science and Technology Medical Centre, Shenzhen, Guangdong, China; ^2^ Shenzhen University Clinical Medical Academy Center, Shenzhen University, Shenzhen, China

**Keywords:** thyroid cancer, ferroptosis, mechanisms, therapeutic targets, reactive oxygen species

## Abstract

Thyroid cancer (TC) represents the most prevalent malignancy within the endocrine system. In recent years, there has been a marked global increase in the incidence of thyroid cancer, garnering substantial scientific interest. Comprehensive investigations into the pathogenesis of TC have identified a significant association with ferroptosis, a newly characterized form of cell death mediated by iron ions. Distinct from apoptosis, necrosis, and autophagy, ferroptosis is characterized by the accumulation of lipid peroxides and reactive oxygen species, culminating in cellular damage and death.Recent research has elucidated a connection between ferroptosis and the initiation, progression, and treatment of thyroid cancer. These findings underscore the significance of ferroptosis in thyroid cancer and offer valuable insights into the development of novel therapeutic strategies and precise predictive markers. The unique mechanisms of ferroptosis present opportunities for targeting treatment-resistant thyroid cancers. Consequently, the regulation of ferroptosis may emerge as a novel therapeutic target, potentially addressing the limitations of current treatments. Moreover, elucidating the molecular mechanisms underpinning ferroptosis in thyroid cancer may facilitate the identification of novel biomarkers for early detection and prognostication. This review endeavors to synthesize the extant knowledge regarding the role of ferroptosis in thyroid cancer, examine potential therapeutic implications, and propose future research trajectories to enhance the understanding and clinical application of ferroptosis.

## 1 Introduction

Thyroid cancer represents the most frequently diagnosed malignancy within the endocrine system and encompasses several subtypes, including papillary thyroid cancer (PTC), follicular thyroid cancer (FTC), medullary thyroid cancer (MTC), and anaplastic thyroid cancer (ATC). Notably, PTC and FTC are collectively referred to as differentiated thyroid carcinomas (DTCs) (1). The standard treatment approach for most thyroid cancers involves surgical resection, radioactive iodine therapy, and thyroid-stimulating hormone suppression (2). Despite these interventions, a subset of patients may experience postoperative recurrence and metastasis, resulting in a compromised prognosis (3, 4). Conversely,ATC remains particularly challenging due to its poor prognosis. Therefore, the continuous exploration of effective treatments underscores the urgent need for novel therapeutic strategies to improve outcomes for all thyroid cancer patients.

Ferroptosis, an iron-dependent form of cell death characterized by the accumulation of ferrous iron ions and lipid peroxidation, is increasingly recognized for its role in a spectrum of diseases (5). Recent studies suggest that ferroptosis may be implicated in various types of cancers, including thyroid cancer (4). This process, marked by the oxidation of cellular membrane lipids, culminates in cell death. Recent studies highlight the relationship between ferroptosis and thyroid cancer, emphasizing its potential impact on disease incidence, progression, and overall patient prognosis (6). These findings have generated interest in ferroptosis as a viable target for novel therapeutic interventions in thyroid cancer and related conditions. Research on ferroptosis in thyroid cancer indicates its promising potential as a therapeutic target (7, 8), suggesting that modulation of this pathway could provide new avenues for intervention. A comprehensive elucidation of the mechanisms underlying ferroptosis and its specific implications in thyroid cancer continues to be an area of active research. The investigation into ferroptosis inhibitors as prospective therapeutic agents underscores their potential to mitigate ferroptosis-induced cell death by obstructing the accumulation of ferrous iron ions and inhibiting lipid peroxidation processes.

This review explores the recent advancements and challenges in ferroptosis research as it pertains to thyroid cancer. We will discuss the signaling pathways involved in ferroptosis and examine the potential of ferroptosis-based therapies to enhance current treatment approaches, offering new strategies to combat thyroid cancer. Additionally, the interaction between ferroptosis and tumor biology will be scrutinized, with an emphasis on the latest research breakthroughs in this domain. Furthermore, this study will critically examine the potential advantages and constraints of incorporating ferroptosis-based methodologies into therapeutic strategies for thyroid cancer. Additionally, it will explore prospective avenues for future research in this domain.

## 2 Thyroid cancer

Thyroid cancer is a malignancy originating from thyroid tissue (9). It can be categorized into four pathological types based on the tissue of origin. DTC, which encompasses PTC and FTC, constitutes approximately 95% of all thyroid malignancies. MTC and ATC account for 3–4% and 1–2% of cases, respectively. In recent years, there has been a significant increase in the global incidence of thyroid cancer (10). According to the 2023 U.S. Epidemiological Survey, thyroid cancer ranks as the fifth most common malignancy among women (11). Recent epidemiological data indicate a twenty-fold increase in the incidence of thyroid cancer among women in Turkey in 2016 compared to the year 2000. Furthermore, thyroid cancer has ascended from the seventh to the third most common malignancy, with a notable trend toward affecting younger individuals (12). According to the 2022 White Paper on Shenzhen Residents’ Health, thyroid cancer accounts for 20.39% of all malignant tumors, rendering it the most prevalent malignancy among women. The rise in thyroid cancer incidence, especially among younger demographics, has led to a reduction in workforce participation and an escalation in public health expenditures, among other societal challenges. Consequently, this has adversely affected the quality of life of the patients.

The onset and progression of thyroid cancer are influenced by a multitude of factors, encompassing genetic mutations, environmental influences, aberrant angiogenesis, and immune system anomalies (13, 14). Current research underscores the pivotal role of the RET and mutated BRAF genes in the progression and metastatic dissemination of thyroid cancer (13). Furthermore, the abnormal activation of the MAPK and PI3K signaling pathways is critically implicated in tumor development (14). Exposure to ionizing radiation and radioactive dust during childhood and adolescence have been established as confirmed risk factors for thyroid cancer (15). Furthermore, aberrant angiogenesis and alterations in the tumor immune environment significantly influence the pathogenesis of thyroid cancer (16).

The primary therapeutic approaches for thyroid cancer encompass surgical intervention, thyroid-stimulating hormone (TSH) suppression, radionuclide therapy, and targeted therapy. Standard treatment protocols generally yield a favorable prognosis for the majority of patients with DTC. Nonetheless, a subset of patients may experience recurrence and metastasis, accompanied by progressive dedifferentiation during the course of treatment. This dedifferentiation contributes to resistance against iodine therapy and targeted therapies, ultimately culminating in a poor prognosis (3, 4). Additionally, ATC constitutes merely 1%-2% of all thyroid cancer cases, it is associated with a dismal 1-year survival rate of only 20% (17, 18). In recent years, the development of targeted therapies for radioactive iodine-refractory differentiated thyroid cancer (RAIR-DTC) has progressed rapidly, addressing a previously unmet need within the therapeutic landscape. However, the overall efficacy of these treatments remains suboptimal due to challenges such as drug resistance and significant adverse reactions. Ensuring drug efficacy while minimizing toxicity and mitigating drug resistance continues to be a critical challenge in this field (19). Therefore, enhancing the efficacy of treatment in these critical cases is essential for improving the overall prognosis of patients with thyroid cancer.

## 3 Ferroptosis

### 3.1 Definition of ferroptosis

**Figure 1:**
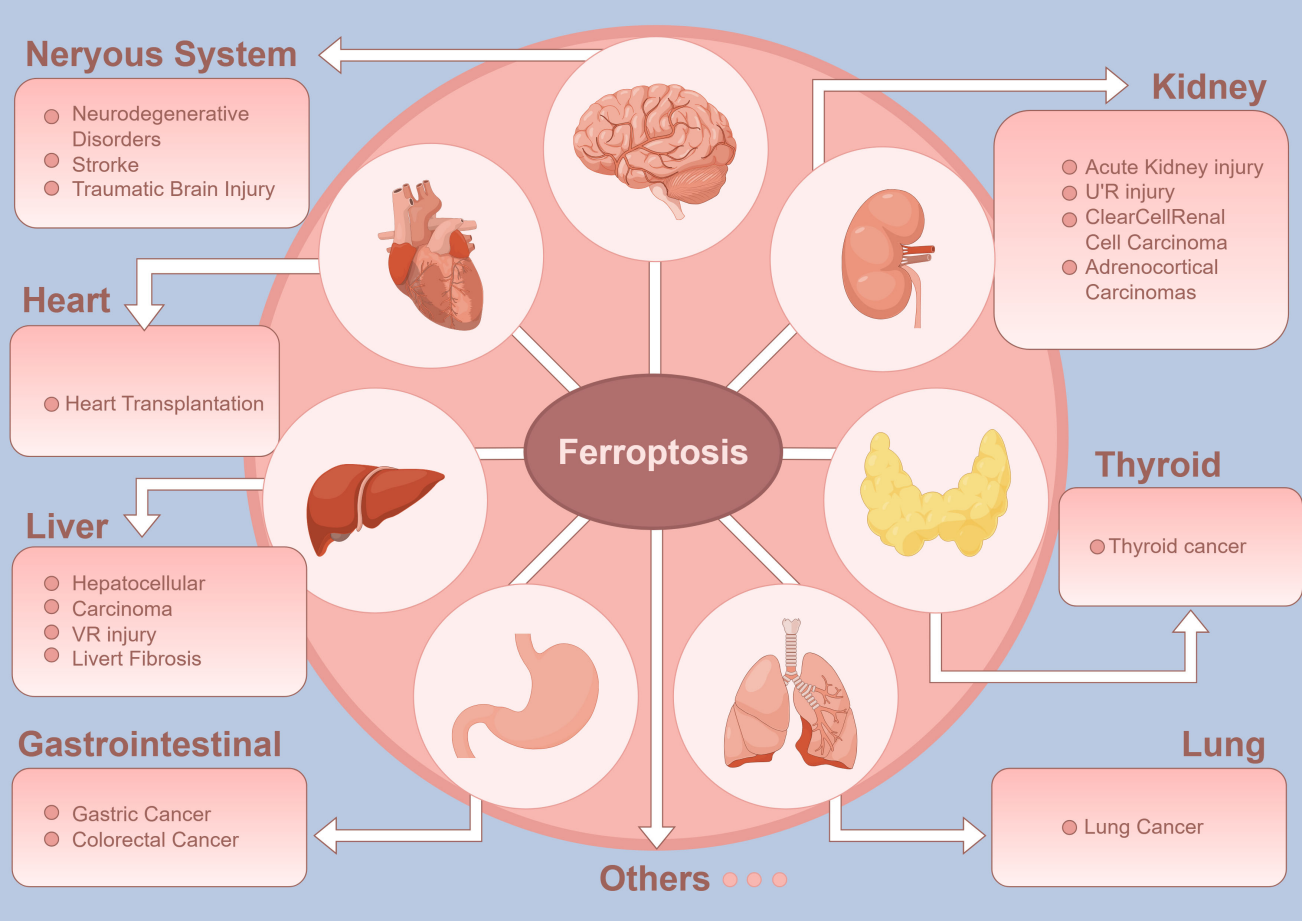
Ferroptosis and its impact on thyroid cancer and other diseases: This diagram highlights ferroptosis, a unique iron-dependent programmed cell death pathway, and its importance in the development and progression of thyroid cancer alongside its impact on various other diseases. This intricate process underscores the therapeutic potential of manipulating ferroptosis in thyroid cancer, providing new avenues for treatment strategies against this and other related diseases.

In 2012, Dr. Dixon’s research group at Columbia University introduced the concept of ferroptosis, a form of programmed cell death closely associated with iron ions and distinct from apoptosis, necrosis, and autophagy (5). The primary mechanism of ferroptosis involves three key steps. The initial step is characterized by the significant accumulation of divalent iron or iron oxygenase esters on the cell membrane. This enzyme catalyzes the high expression of unsaturated fatty acids, resulting in lipid peroxidation (20–22). The subsequent step transpires when cellular cystine transport proteins are inhibited, for instance, by erastin, leading to a reduction in intracellular glutathione levels. This reduction results in the inactivation of glutathione peroxidase 4 (GPX4), thereby permitting the accumulation of lipid peroxides. Upon reaching a critical threshold, cells undergo cell death (5, 23, 24). GPX4 inhibitors, such as RasLethal3 (RSL3), can directly initiate this process by downregulating GPX4 expression, consequently elevating intracellular lipid peroxide levels (5, 25). Ferroptosis induces nuanced modifications within the mitochondria, such as mitochondrial membrane contraction, diminution or loss of mitochondrial cristae, and disruption of the outer membrane. These mitochondrial changes, coupled with the oxidative degradation of cell membrane lipids, collectively facilitate cell death. Concurrent with cell death, there is a significant release of divalent iron ions (26–28). In summary, the mechanism of cellular ferroptosis is intricate and encompasses multiple factors influenced by various substances. Central to this process are divalent iron ions, lipid peroxides, and glutathione (**Figure 1**).

### 3.2 Ferroptosis process

Ferroptosis is contingent upon three primary conditions: the accumulation of divalent iron, the accumulation of lipid peroxides, and the depletion of glutathione. The presence of these conditions initiates a cascade of reactions that result in elevated intracellular peroxide levels, oxidation of cellular membrane lipids, and ultimately, cell death (5). Ferroptosis represents a distinct modality of regulated cell death characterized by iron-dependent oxidative damage and disruption of cellular membranes (22). Furthermore, this process involves the interplay of several key proteins, including p53, voltage-dependent anion channels (VDACs), and cystine/glutamate antiporters (system XC-), which collectively contribute to the regulation and execution of ferroptosis.

**Figure 2:**
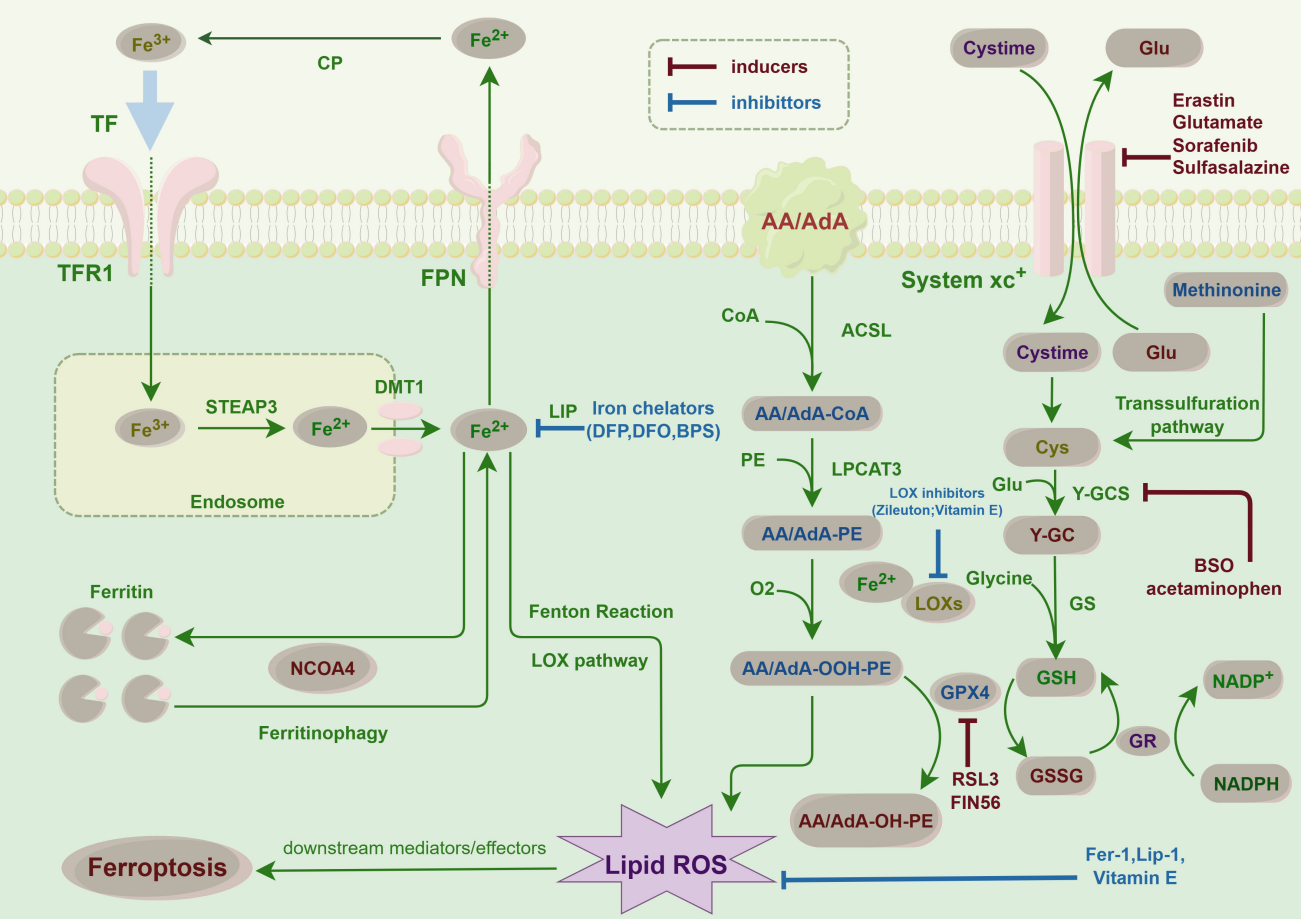
Ferroptosis pathways in cancer cells: This figure illustrates the key molecular pathways leading to ferroptosis, an iron-dependent form of cell death, in thyroid cancer. Central to ferroptosis is the accumulation of iron, which catalyzes the formation of lipid peroxides, causing lethal damage to cells. The diagram highlights critical steps such as iron uptake and storage, lipid peroxidation, and the role of glutathione and GPX4 in detoxifying peroxides. This finding highlights the balance between proferroptotic inducers, such as erastin, which inhibits cystine uptake, and antiferroptotic mechanisms, including iron chelators and antioxidants. This balance is crucial for the survival or death of thyroid cancer cells, suggesting potential therapeutic targets for modulating ferroptosis in cancer treatment.

Ferroptosis represents a unique modality of cell death characterized by an imbalance between the production and degradation of intracellular lipid reactive oxygen species, culminating in oxidative cell death (29). A variety of compounds are capable of inducing ferroptosis through diverse pathways. These upstream pathways ultimately influence the activity of glutathione peroxidases (GPXs), thereby diminishing the antioxidant capacity of cells. This reduction in antioxidant defense facilitates the accumulation of lipid peroxidation and lipid reactive oxygen species, consequently inducing ferroptosis (30). For instance, the pathway facilitated by system XC- is of critical importance. Inhibition of system XC- results in diminished cystine uptake, a subsequent reduction in GPXs activity, and ultimately induces ferroptosis (31). Additionally, the activation of the p53 gene within the p53-mediated pathway can inhibit cystine uptake by downregulating system XC-, leading to decreased GPXs activity and an accumulation of lipid reactive oxygen species, thereby promoting ferroptosis (32). Furthermore, there exist mechanisms that directly inhibit GPX4. For instance, RSL3 specifically targets GPX4, resulting in a reduction of cellular antioxidant capacity, an elevation in lipid reactive oxygen species, and the initiation of ferroptosis (33). Additionally, voltage-dependent anion channels (VDACs) influence mitochondrial function, leading to the release of oxidizing agents and culminating in oxidative cell death (34). Other pathways, including those regulated by sulfur transfer, heme oxygenase 1, and transferrin, among others, are implicated in the regulation of ferroptosis. The oxidation of polyunsaturated fatty acids (PUFAs) within cell membrane lipids is pivotal for the generation of lipid reactive oxygen species, a process that is expedited by the presence of iron ions, thereby promoting ferroptosis in cells (35). Given the essential role of iron in ferroptosis, various iron chelators have been shown to inhibit this form of cell death. However, the specific effects of these chelators can differ, particularly in their influence on the production of reactive oxygen species (36). Therefore, elucidating the mechanism of ferroptosis will facilitate subsequent investigations into the factors influencing this process in thyroid cancer cells (**Figures 1**, **2**).

### 3.3 Factors involved in the process of ferroptosis

#### 3.3.1 Factors facilitating

Ferroptosis can be induced by a multitude of factors, including iron accumulation, oxidative stress, and the inhibition of gene expression associated with iron metabolism. Elevated concentrations of free iron ions are capable of initiating cell death. Iron accumulation may arise from prolonged excessive iron intake, disorders of iron metabolism, and other conditions, such as hereditary hemoglobinopathies (37).

Biological oxidation transpires when the generation of oxygen free radicals within cells surpasses the cell’s antioxidant capacity. These oxygen free radicals interact with iron ions to form highly reactive oxides, thereby initiating cellular ferroptosis (38). Furthermore, members of the iron regulatory protein (IRP) family play a pivotal role in modulating the expression of genes involved in cellular iron metabolism (39). The suppression of these gene regulators can result in the intracellular accumulation of iron ions, thereby facilitating the onset of ferroptosis.

Furthermore, specific test agents, including elastin and RSL3 [(1S,3R)-RSL3, an inhibitor of GPX4 that reduces GPX4 protein expression], as well as approved pharmaceuticals such as sorafenib, sulfasalazine, statins, and artemisinin, along with ionizing radiation and cytokines such as IFNγ and TGFβ1, have been demonstrated to induce ferroptosis (40, 41) (**Figure 2**). Several compounds have been shown to modulate ferroptosis in thyroid cancer, including sorafenib, which inhibits oxidative stress pathways and induces ferroptosis (42). Studies have also examined elastin and RSL3, both of which have demonstrated potential to induce ferroptosis in thyroid cancer cell lines, offering new therapeutic avenues (43).

#### 3.3.2 Factors inhibiting

Several factors can impede the induction of ferroptosis, including iron deficiency, elevated ferritin expression, antioxidant systems, and the presence of iron chelators. Ferroptosis is initiated by the intracellular concentration of free iron ions, which remains low under conditions of iron deficiency. Iron deficiency can result from various causes, such as inadequate dietary intake, malabsorption, or chronic hemorrhage. Elevated levels of ferritin, an intracellular iron storage protein, can sequester free iron ions, thereby inhibiting ferroptosis (44). Consequently, elevated ferritin expression can mitigate intracellular free iron ion levels, thereby inhibiting ferroptosis. Furthermore, several studies have identified dysregulation of iron metabolism in patients with high-grade serous ovarian cancer. An upregulation of transferrin receptor 1 may result in a downregulation of the iron ion pump and membrane ferrotransporter expression, potentially facilitating tumor growth and metastasis (39).

Intracellular antioxidant systems are capable of neutralizing oxygen free radicals, thereby mitigating cellular damage induced by oxidative stress. A robust antioxidant capacity within cells may inhibit the initiation of ferroptosis (45). Iron chelating agents, which are molecules that sequester free iron ions and render them inactive, can effectively decrease intracellular free iron ion concentrations, thus preventing the onset of ferroptosis (46) (**Figure 2**).

## 4 Role of ferroptosis in cancer

Since its identification as a distinct form of oxidative cell death characterized by iron accumulation and lipid peroxidation, ferroptosis has garnered significant global interest. This process is critically implicated in the pathogenesis of a variety of diseases, such as tumors, ischemia/reperfusion injury, neurological disorders, and renal injury. Consequently, ferroptosis presents a promising avenue for novel therapeutic interventions.

Recent studies indicate that ferroptosis is critically involved in the initiation, progression, proliferation, metastasis, and therapeutic response of tumors. Ferroptosis exhibits dual roles in tumorigenesis, capable of both promoting and inhibiting tumor growth. For instance, in pancreatic ductal adenocarcinoma, the enzyme CYP2J2 inhibits ferroptosis by generating epoxyeicosatrienoic acids (EETs), which consequently enhance tumor survival. EETs suppress ferroptosis through a PPARγ-dependent mechanism by upregulating GPX4 levels, thereby augmenting the survival of tumor cells (47). These findings indicate that tumor cells may evade ferroptosis by modulating their iron metabolism, thereby supporting their proliferation and survival. Furthermore, ferroptosis has the potential to inhibit tumor growth by augmenting immune responses during tumorigenesis. Conversely, it may also contribute to tumor development by promoting the release of damage-associated molecular patterns (48).

Current research has predominantly concentrated on the tumor-inhibitory effects of ferroptosis. Ferroptosis has been demonstrated to interact with several tumor suppressor factors, such as p53 and BAP1 (49, 50). For instance, p53 can potentiate ferroptosis by modulating cellular iron metabolism and influencing the production of reactive oxygen species (ROS), which are integral to the ferroptosis process (49). Specifically, p53 regulates the import and export of iron, the stability of intracellular iron, and iron storage, all of which are essential for the regulation of ferroptosis (49). Furthermore, p53 influences lipid peroxidation and ROS levels within the cell, both of which are integral to the mechanism of ferroptosis (51). BAP1 facilitates ferroptosis by downregulating the expression of solute carrier family 7 member 11 (SLC7A11), a cystine/glutamate antiporter. This repression results in decreased levels of reduced glutathione and a consequent reduction in the antioxidant capacity of the cells, leading to the accumulation of lipid-ROS and the induction of ferroptosis (52). This evidence indicates that ferroptosis may be implicated in the resistance to cancer development (49, 50). Furthermore, ferroptosis has been demonstrated to contribute to tumor proliferation and metastasis. For instance, the inhibition of ferroptosis in liver cancer cells has been observed to enhance tumor growth and metastasis, whereas the induction of ferroptosis can mitigate these processes (53). Recent research indicates that ubiquitin-specific peptidase 10 (USP10) facilitates the proliferation and metastasis of tumor cells by inhibiting ferroptosis in thyroid cancer cells (54). It is crucial to adopt a balanced and objective tone when examining the impact of ferroptosis on tumor progression.

Ferroptosis is increasingly recognized as a pivotal mechanism in cancer therapy. Emerging research indicates that ferroptosis may influence the efficacy of chemotherapy, radiotherapy, and immunotherapy (55). Specifically, in the treatment of thyroid cancer, the induction of ferroptosis is posited to hold substantial therapeutic promise (56). Consequently, augmenting ferroptosis in thyroid cancer cells could constitute a viable therapeutic approach for addressing certain refractory thyroid cancers.

In summary, research on ferroptosis has demonstrated considerable therapeutic potential across various cancer types. Advancing our comprehension of the mechanisms underlying ferroptosis and examining its interactions with other treatment modalities may unveil novel directions and strategies for future cancer therapies.

## 5 Relationship between ferroptosis and thyroid cancer

Recent research has elucidated a close association between ferroptosis and cancer progression, metastasis, and drug resistance. Consequently, the investigation of ferroptosis in oncological contexts, including thyroid cancer, represents a critical research trajectory encompassing diverse molecular mechanisms and therapeutic strategies. Contemporary studies have concentrated on the modulation of specific signaling pathways or molecular targets to either induce or inhibit ferroptosis, thereby offering novel insights for the treatment of thyroid cancer.

### 5.1 Ferroptosis and coding genes

Recently, researchers have identified genes associated with ferroptosis in thyroid cancer through the utilization of published databases. Specifically, Shi et al. investigated the relationships between various types of thyroid cancer and ferroptosis-related genes (FRGs). They developed a novel ferroptosis-related gene model consisting of 10 genes using the Least Absolute Shrinkage and Selection Operator (LASSO) regression model, which has the potential to predict the prognosis of patients with papillary thyroid cancer (6). Moreover, Yang et al. conducted an analysis of mRNA expression profiles and clinical data from patients with PTC, identifying significant differences in FRGs between tumor tissues and adjacent normal tissues. Their study revealed that, in the majority of tumors, the expression levels of these genes were elevated compared to the adjacent normal tissues (57). These findings imply the involvement of multiple coding genes in the ferroptosis process within thyroid cancer cells, thereby necessitating further investigation into their specific roles and impacts.

Furthermore, researchers identified that ETS variant transcription factor 4 (ETV4) facilitates ferroptosis through the downregulation of SLC7A11, a specific amino acid transporter. *In vitro* studies revealed that ETV4 is overexpressed in thyroid cancer tissues. Silencing ETV4 resulted in the inhibition of proliferation and cell cycle progression in papillary thyroid cancer cells, and it suppressed tumor growth by inducing ferroptosis. *In vivo* experiments corroborated that the downregulation of ETV4 leads to reduced expression of SLC7A11, thereby inhibiting tumor development (58, 59). These findings suggest potential biomarkers and therapeutic targets for the treatment of PTC. In 2022, another researcher identified that the fat mass and obesity-associated protein (FTO) can bind to the potential m6A site of the SLC7A11 3’ untranslated region (3’ UTR). This study demonstrated that the overexpression of m6A demethylation inhibits the expression of SLC7A11, thereby promoting ferroptosis in PTC and suppressing PTC growth (60). This study extends prior research on ferroptosis in thyroid carcinoma, offering novel research directions and potential therapeutic targets. Additionally, the findings corroborate the expression and functional significance of SLC7A11 in thyroid carcinoma, underscoring its pivotal role in the ferroptosis process.

The most recent study conducted in 2023 demonstrated that SIRT6, an NAD+-dependent histone deacetylase, can induce NCOA4-dependent autophagy to degrade ferritin through overexpression. This process results in elevated intracellular Fe2+ levels, thereby sensitizing cells to ferroptosis. The role of SIRT6 in this mechanism was further corroborated by *in vivo* experiments (61). These findings indicate that SIRT6 may serve as a potential therapeutic target for thyroid cancer. However, recent research has demonstrated that ALKBH5, an RNA demethylase homologous to AlkB, can impede the progression of thyroid cancer by inducing ferroptosis in thyroid cancer cells via the TIAM1-Nrf2/Heme Oxygenase 1 (HO-1) axis, as evidenced by both *in vitro* and *in vivo* studies. The results demonstrated a reduction in ALKBH5 expression and an elevation in T-cell lymphoma invasion and metastasis 1 (TIAM1) expression in thyroid cancer. ALKBH5 modulates TIAM1 expression via m6A modification, thereby promoting ferroptosis in thyroid cancer cells (62). Additionally, Chen et al. reported an aberrant increase in HO-1 expression in thyroid cancer, which led to decreased cell viability and the activation of ferroptosis signaling. Curcumin was found to further enhance the ferroptosis pathway by upregulating HO-1 expression, consequently inhibiting thyroid cancer growth (63).

These findings suggest that ferroptosis plays a crucial role in the proliferation of thyroid cancer cells. Targeting specific mechanisms or pathways through pharmacological agents or reagents that induce ferroptosis may enhance the expression of associated genes or activate relevant pathways. This strategy holds promise for inhibiting the growth of thyroid cancer cells and achieving therapeutic outcomes.

### 5.2 Ferroptosis and non-coding genes

Ferroptosis is associated with both coding and non-coding genes. Recent investigations have identified the circular RNA Circ_0067934 as a significant contributor to the ferroptosis process in thyroid cancer cells, where it modulates the miR-545-3p/SLC7A11 signaling pathway. Additionally, Chen et al. reported that the circular RNA circKIF4A, which is overexpressed in PTC, enhances cancer cell proliferation and migration by inhibiting apoptosis (64). A separate investigation demonstrated that the RNA circKIF4A facilitates the malignant progression of PTC through its interaction with miR-1231, subsequently upregulating the expression of GPX4, an antioxidant protein (65). These results offer novel insights into thyroid cancer treatment and propose potential therapeutic strategies for future clinical interventions.

In 2022, Qin et al. conducted an analysis of the TCGA THCA dataset and subsequently developed a prognostic model predicated on five long non-coding RNAs (lncRNAs). The model demonstrated high accuracy in predicting the survival outcomes of thyroid cancer patients. These results imply that an upregulation of specific lncRNAs may enhance the prognosis for individuals with thyroid cancer. Furthermore, these lncRNAs appear to mediate anti-tumor immune responses and could serve as potential therapeutic targets for the treatment of thyroid cancer (66). Furthermore, Lin et al. formulated a prognostic model utilizing ferroptosis-related long non-coding RNAs (lncRNAs) to predict the survival outcomes of thyroid cancer patients. This study included an analysis of immune cell infiltration and immune gene expression within the tumor immune microenvironment (67). In 2023, researchers employed statistical methodologies to examine the association between ferroptosis-related lncRNAs and ferroptosis in PTC. They subsequently developed a prognostic model based on these ferroptosis-related lncRNAs (FRLs) to evaluate their prognostic significance in patients with PTC (68).

The results suggest that non-coding genes may play a role in promoting ferroptosis in thyroid cancer cells, thereby offering potential therapeutic advantages. Nonetheless, further experimental investigations are essential to substantiate these findings, necessitating continued exploration in this area by future researchers.

### 5.3 Ferroptosis and the immune microenvironment

**Figure 3:**
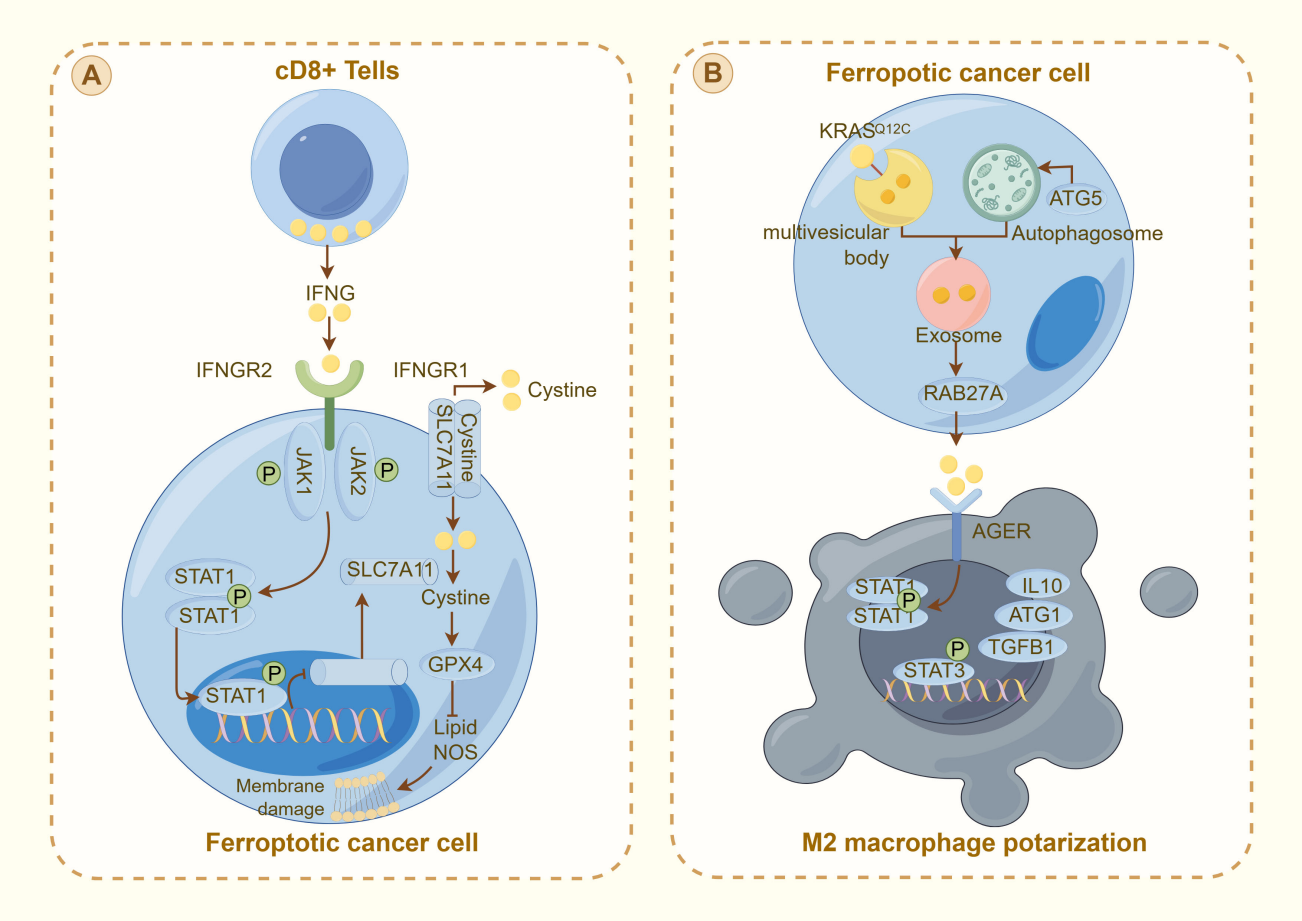
Interaction between ferroptosis and the immune response in cancer cells: **(A)** illustrates the activation of ferroptosis in cancer cells by CD8+ T cells through the IFNγ pathway. IFNG released from CD8+ T cells binds to its receptor (IFNGR1 and IFNGR2) on the cancer cell surface, activating the JAK-STAT pathway. This activation downregulates SLC7A11, reducing cystine uptake and promoting GPX4 inactivation, leading to lipid peroxidation and membrane damage characteristic of ferroptosis. **(B)** depicts ferroptotic cancer cells influencing the tumor microenvironment. The cell releases exosomes through RAB27A, signaling to nearby M2 macrophages. This interaction, which is mediated by AGER, modulates the immune response through STAT signaling, influencing the production of cytokines (e.g., IL10 and TGFβ1) and contributing to M2 macrophage polarization. This mechanism illustrates the complex interplay between ferroptosis and immune evasion in thyroid cancer, highlighting potential therapeutic targets to increase cancer treatment efficacy by modulating the TME.

In addition to thyroid cancer cells, thyroid cancer tissues encompass lymphocytes, immune cells, fibroblasts, and various extracellular matrix components, collectively constituting the tumor microenvironment (TME), which influences tumor growth, invasion, and therapeutic response (69). Qin et al. identified differentially expressed genes (DEGs) between thyroid tumors and normal tissues, demonstrating a significant association between ferroptosis and the TME, particularly with tumor immune cells (70). Additionally, Ren et al. developed a prognostic prediction model incorporating eight ferroptosis-related non-coding genes. Utilizing Lasso-Cox regression analysis, this model stratifies patients into high-risk and low-risk categories based on the expression levels of these genes (71). These findings underscore the significant association between ferroptosis and the immune microenvironment, emphasizing the critical interplay between ferroptosis-related genes and immune responses within the tumor milieu (**Figure 3**).

Substantial advancements have been achieved in the study of the tumor immune microenvironment and genetic research. In 2021, Ming et al. developed a predictive model utilizing ferroptosis-related genes linked to the immune microenvironment to forecast clinical outcomes in thyroid cancer patients. Furthermore, Fan et al. (72) demonstrated that modifications in the immune microenvironment significantly influence the ferroptosis of thyroid cancer cells (73). This study provides additional evidence supporting the strong association between ferroptosis and the immune microenvironment. In 2023, Lin et al. highlighted the pivotal role of immune cells and signaling molecules within the immune microenvironment in modulating the ferroptosis process in thyroid cancer cells. Furthermore, they reported that FRGs are linked to tumor immunity and drug resistance, thereby serving as significant indicators of the clinical prognosis of cancer patients (72). Research has demonstrated that a high level of M2 macrophage infiltration is frequently correlated with a poor prognosis. Conversely, an elevated density of infiltrating dendritic cells may be indicative of a more favorable prognosis (67). These observations suggest that the immune microenvironment exerts a substantial influence on the regulation of ferroptosis in thyroid cancer cells, thereby highlighting a promising avenue for future investigation.

### 5.4 Ferroptosis and biological oxidation

The process of biological oxidation is intricately associated with ferroptosis in thyroid cancer, as evidenced by the findings of Shi et al. (6) and Yang et al. (57). Their research elucidated the modulation of FRGs and its impact on the oxidative balance within cancer cells, thereby presenting a novel perspective for therapeutic development. By targeting specific pathways that regulate lipid peroxidation and reactive oxygen species, researchers could potentially develop more effective treatments for thyroid cancer.

GPX4 is an intracellular selenoprotein antioxidant enzyme responsible for the elimination of membrane lipid hydrogen peroxide products, thereby mitigating oxidative stress (74). It employs reduced glutathione to catalyze the reduction of hydroperoxides, organic peroxides, and lipid peroxides, thereby maintaining intracellular oxidative homeostasis (23). In contrast to other members of the GPX family, GPX4 exhibits a monomeric structure. It facilitates the reduction of lipid peroxides within the cell membrane with minimal dependence on glutathione as a reducing substrate (75). GPX4 plays a pivotal role in regulating the ferroptosis process and has the potential to affect tumor growth by modulating ROS levels.

Numerous studies have demonstrated the critical role of GPX4 in the ferroptosis of thyroid cancer cells. GPX4 expression is markedly elevated in thyroid cancer tissues. For instance, Chen et al. reported a significant upregulation of GPX4 expression in thyroid cancer, which was closely associated with tumor malignancy and patient prognosis. Furthermore, the overexpression of GPX4 inhibits ferroptosis, thereby promoting the proliferation and metastasis of thyroid cancer (76).

In 2019, Sekhar et al. demonstrated that GPX4 inhibitors are capable of inducing ferroptosis in PTC cells, thereby significantly inhibiting their proliferation. This effect was notably more pronounced in cells harboring co-mutations in BRAF, RAS, TERT promoters, and PIK3CA (77). These results imply a reciprocal relationship between GPX4 inhibition and specific tumor mutational profiles. Furthermore, Pamarthy et al. identified a diaryl ether-derived compound that can downregulate GPX4 expression, subsequently inducing ferroptosis in thyroid cancer cells (78). Furthermore, empirical evidence indicates that RSL3, a small molecule inhibitor, effectively targets GPX4, thereby inducing ferroptosis in PTC cells (79). These observations imply that the induction of ferroptosis in malignant cells holds potential as a therapeutic strategy for inhibiting thyroid cancer progression through the targeted modulation of specific molecules or the inhibition of GPX4 expression and function. Consequently, this domain represents a promising avenue for future research endeavors.

ROS are byproducts of normal aerobic metabolism within the body. These species encompass peroxides, superoxides, hydroxyl radicals, singlet oxygen, and α-oxygen. It is crucial to employ these precise scientific terms consistently throughout the text. The Fenton reaction, which involves iron and hydroperoxides, generates highly reactive and toxic hydroxyl radicals (80). ROS produced through the Fenton reaction and subsequent lipid peroxidation are integral to the process of ferroptosis in thyroid cancer cells (81). Various oxidative and antioxidant mechanisms work in concert to modulate lipid peroxidation during ferroptosis in thyroid cancer cells (82). The involvement of ROS in the regulation of ferroptosis within these cells is of paramount importance. The generation of ROS is contingent upon the activity of NADPH oxidase and the mitochondrial respiratory chain, both of which facilitate lipid peroxidation through the actions of lipoxygenase or cytochrome P450 reductase (80).

For instance, a study conducted in 2021 demonstrated that vitamin C can induce autophagy, degrade ferritin, release free iron, and initiate the Fenton reaction to generate ROS (83). This cascade sustains lipid peroxidation and culminates in ferroptosis in ATC cells (84). These findings propose potential therapeutic strategies for the treatment of ATC.

Furthermore, a study conducted in 2022 demonstrated that anlotinib exerts its antitumor effects on ATC via the autophagy-ferroptosis signaling pathway. This study also found that the use of autophagy inhibitors could potentiate anlotinib-induced ferroptosis and enhance its antitumor efficacy (85). Subsequent investigations have demonstrated that anlotinib induces ferroptosis by downregulating ferroptosis-related targets, including transferrin, HO-1, FTH1, FTL, and GPX4, while concurrently increasing ROS levels. In a retrospective study conducted in 2023, Inoue M et al. reported that human mercury (HMA) facilitates the accumulation of intracellular ROS in DTC cells through lipid peroxidation. This finding suggests that HMA inhibits the survival, proliferation, and migration of undifferentiated thyroid cancer cells, thereby promoting ferroptosis in DTC and impeding thyroid cancer progression (86, 87).

All of these studies employ ROS as the foundational element to investigate the mechanisms and processes underlying ferroptosis in thyroid cancer cells. The primary objective of these investigations is to identify effective therapeutic targets. Through a comprehensive examination of the relationship between ROS and ferroptosis in thyroid cancer cells, these studies have yielded novel insights and conceptual advancements for the treatment of thyroid cancer. Future research endeavors will persist in exploring the role of ROS, with the aim of furnishing a more robust theoretical framework and practical guidance for the clinical management of thyroid cancer.

## 6 Discussion

The global incidence of thyroid cancer, a common endocrine malignancy, is on the rise, particularly among younger populations. This trend highlights an urgent imperative for the scientific and medical communities to devise innovative therapeutic strategies. This review examines ferroptosis, a recently identified form of cell death mediated by iron ions, and summarizes its advancements in the context of thyroid cancer research.

**Figure 4:**
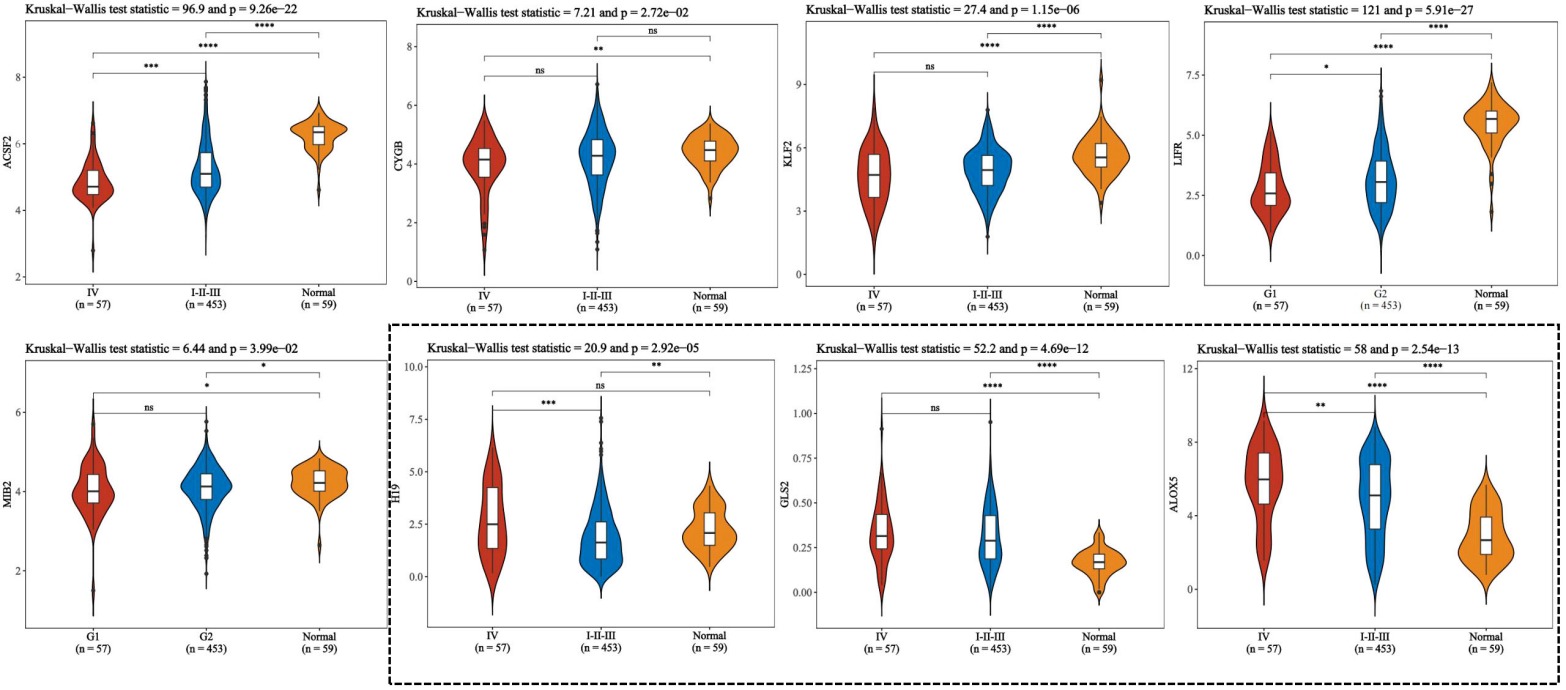
To compare the expression of ferroptosis-related genes between stage IV and stage I to III of thyroid cancer according to pTNM staging. This figure presents violin plot and box plot of expression distribution of ferroptosis-related genes in thyroid cancer stage IV, I to III, and normal tissues. The abscissa represents the different sample groups, and the ordinate represents the expression distribution of the gene. Different colors represent different groups. Asterisks in the upper left corner represent significant p values, *p < 0.05, **p < 0.01, ***p < 0.001, **** indicates p< 0.0001, “ns” means “Non-Significant”. The significance of two groups of samples was determined by Wilcoxon test or t-test, while the significance of three groups of samples was determined by Kruskal-Wallis test.

**Figure 5:**
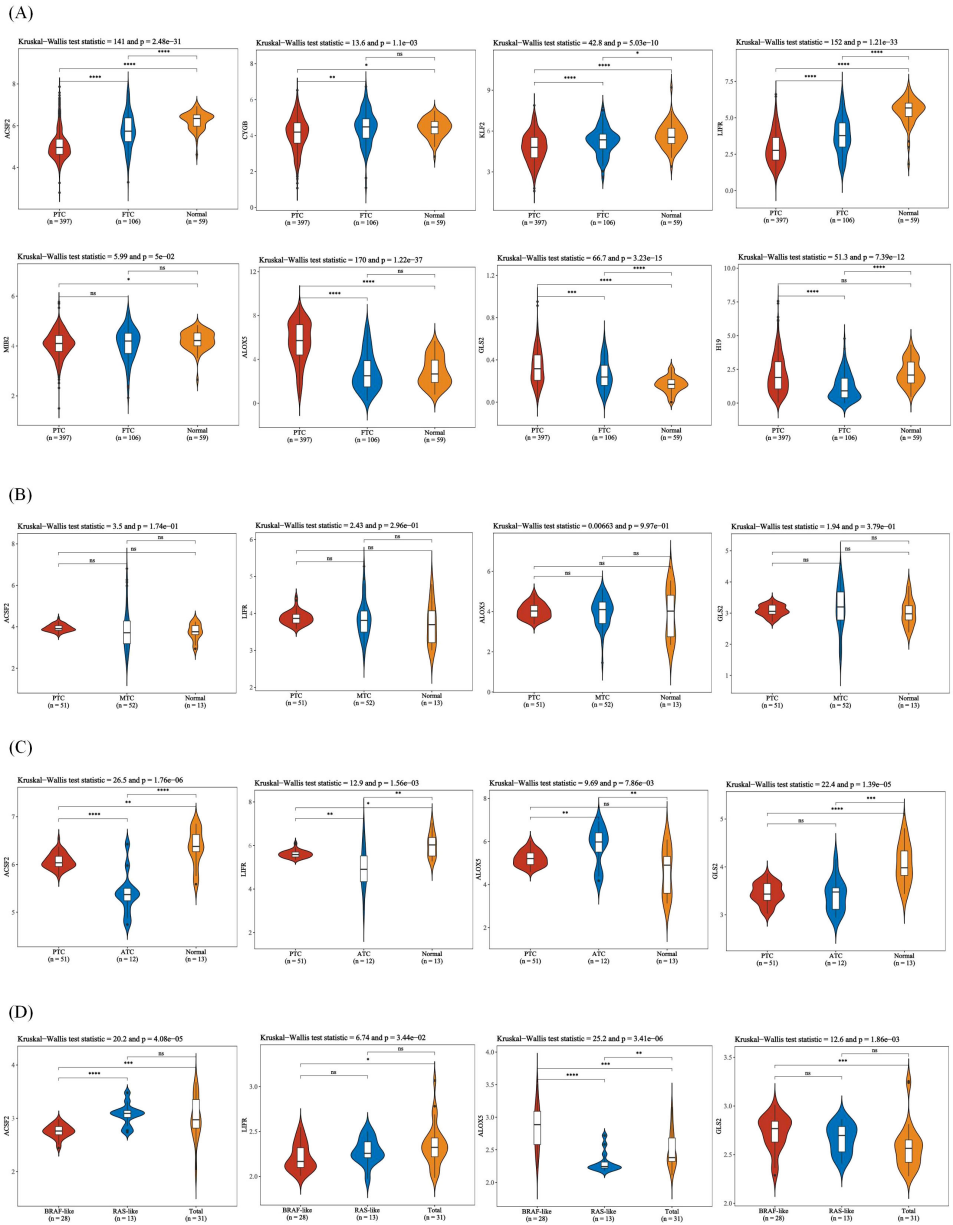
Expression of ferroptosis-related genes in different thyroid cancer types. **(A)** Expression of ferroptosis-related genes in PTC, FTC and normal tissues. **(B)** Expression of ferroptosis-related genes in PTC, MTC and normal tissues. **(C)** Expression of ferroptosis-related genes in PTC, ATC, and normal tissues. **(D)** Expression of ferroptosis-related genes in BRAF-like, RAS-like and overall thyroid cancer. The x-axis represents different sample groups, and the y-axis represents the expression distribution of the gene. Different colors represent different groups. The asterisks in the upper left corner represent the significance p-value, where * indicates p < 0.05, ** indicates p < 0.01, *** indicates p < 0.001 and **** indicates p< 0.0001, “ns” means “Non-Significant”. The number of asterisks represents the degree of significance. The significance of two sample groups is determined by the Wilcoxon test or T-test, while the significance of three sample groups is determined by the Kruskal-Wallis test.

**Figure 6:**
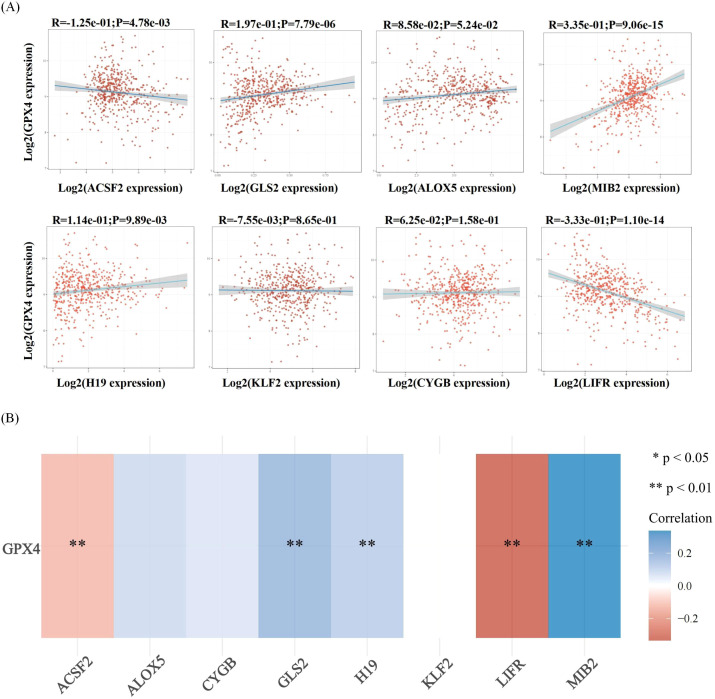
Correlation analysis of ferroptosis-related genes and GPX4 in thyroid cancer. **(A)** The correlations between two genes: The expression correlation of two genes was analysed with Spearman. The abscissa represents the expression distribution of the first gene, and the ordinate represents the expression distribution of the second gene. The density curve on the right represents the trend in distribution of the second gene, the upper density curve represents the trend in distribution of first gene expression. The value on the top represents the correlation p value, correlation coefficient and correlation calculation method. **(B)** The correlations among multiple genes: A heatmap of the correlation between eight genes and GPX4.The abscissa and ordinate represent genes, different colors represent different correlation coefficients (blue represents positive correlation whereas red represents negative correlation), the darker the color, the stronger the relation. Asterisks (*) stand for significance levels, **p < 0.01.

**Figure 7:**
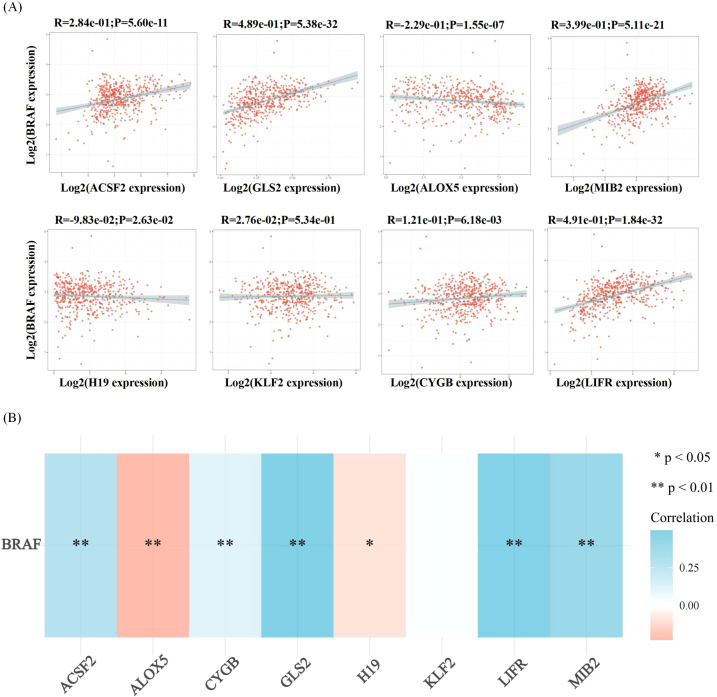
Correlation analysis of ferroptosis-related genes and BRAF in thyroid cancer. Correlation analysis between ferroptosis-related genes and BRAF among the highly expressed genes in thyroid cancer. **(A)** The correlations between two genes: The expression correlation of two genes was analysed with Spearman. The abscissa represents the expression distribution of the first gene, and the ordinate represents the expression distribution of the second gene. The density curve on the right represents the trend in distribution of the second gene, the upper density curve represents the trend in distribution of first gene expression. The value on the top represents the correlation p value, correlation coefficient and correlation calculation method. **(B)** The correlations among multiple genes: A heatmap of the correlation between eight genes and BRAF. The abscissa and ordinate represent genes, different colors represent different correlation coefficients (blue represents positive correlation whereas red represents negative correlation), the darker the color, the stronger the relation. Asterisks (*) stand for significance levels, **p < 0.01, *p < 0.05.

Ferroptosis, characterized by mechanisms and features distinct from traditional apoptosis and autophagy, provides novel insights into the treatment of refractory thyroid cancers. In the realm of thyroid cancer, substantial advancements have been achieved in elucidating the role of ferroptosis (66, 88). Consequently, we performed some bioinformatics analyses using TCGA database, GEO database, and FerrDb database. Comparison of ferroptosis-related genes cataloged in the FerrDb database with thyroid cancer-related genes identified 8 genes potentially associated with siderosis in thyroid cancer (**Table 1**). We compared ferroptosis-related gene expression in thyroid cancer stages IV and I-II-III (according to pTNM stage), ferroptosis-related gene expression in PTC and FTC, MTC, ATC respectively, and ferroptosis-related gene expression in BRAF-like, RAS-like and overall thyroid cancer. We found that most of the ferroptosis-related genes were repressed in stage IV thyroid cancer, and there was no significant difference in the expression of ferroptosis-related genes in other comparisons (**Figures 4**, **5**). Meanwhile, we also analyzed the expression levels of eight genes related to GPX4 and BRAF in thyroid cancer to further elucidate their association with thyroid ferroptosis and prognosis (**Figures 6**, **7**). These findings may provide potential avenues for follow-up studies.

**Table 1:** Associated ferroptosis-related genes in thyroid cancer.

Target gene	Function	Pathway	Refs
ACSF2	driver	ACSF2 promotes ferroptosis by enhancing lipid peroxidation and disrupting iron homeostasis.	(5)
GLS2	driver	GSL2 may increase ferroptosis by elevating glutamate levels, which in turn depletes intracellular glutathione, leading to increased lipid per-oxidation and triggering ferroptosis.	(89)
ALOX5	driver	ALOX5 increases the production of lipid ROS, and the accumulation of these lipid ROS directly contributes to the induction of ferroptosis.	(90)
MIB2	driver	MIB2 negatively regulates GPX4, and the down-regulation of GPX4 leads to increased susceptibility to ferroptosis.	(91)
KLF2	driver	KLF2 negatively regulates GPX4, and the reduction of GPX4 expression increases the likelihood of ferroptosis.	(92)
H19	driver	H19 negatively regulates miR-106b-5p, which in turn negatively regulates ACSL4; as a result, up-regulation of ACSL4 promotes ferroptosis.	(93)
CYGB	driver	CYGB up-regulates p53, which in turn up-regulates YAP1. YAP1 increases ACSL4 expression, leading to the accumulation of lipid ROS, which ultimately promotes ferroptosis.	(94)
LIFR	driver	LIFR up-regulates SHP1, which negatively regulates NF-κB signaling. Reduced NF-κB signaling leads to the up-regulation of LCN2, which in turn inhibits ferroptosis.	(95)

It has also been reported that ferroptosis may be less pronounced in low-risk cancers, such as well-differentiated PTC, because these tumors typically respond well to conventional therapies. However, at advanced stages, especially in aggressive subtypes such as ATC and MTC, ferroptosis may be impaired, resulting in chemoresistance (43). Ferroptosis may play a critical role in the progression of thyroid cancer. In low-risk thyroid cancer, such as well-differentiated PTC, ferroptosis might be less pronounced, as these tumors often respond well to conventional therapies. However, in advanced stages, particularly in aggressive subtypes like ATC and MTC, ferroptosis may be impaired, contributing to chemoresistance. For example, PTC may show different ferroptosis characteristics compared with ATC or MTC, reflecting differences in iron metabolism, oxidative stress response and antioxidant defense system. Specific molecular alterations, such as SLC7A11 overexpression in high-risk tumors, may render advanced tumors more resistant to ferroptosis (42). ATC and relapsed MTC represent highly aggressive and treatment-resistant subtypes of thyroid cancer. Ferroptosis may contribute to their progression by promoting oxidative damage and inhibiting effective cell death mechanisms. Targeting ferroptosis pathways in these cancers could provide novel strategies for overcoming treatment resistance (70). Furthermore, thyroid cancers are classified based on histological and molecular features, such as BRAF-like and RAS-like subtypes, each of which exhibits different molecular features and prognosis. Recent studies have reported differences in metabolic phenotypes among molecular classifications (96). We also conducted correlation analysis, and no significant difference was found (**Figures 5**).

**Figure 8:**
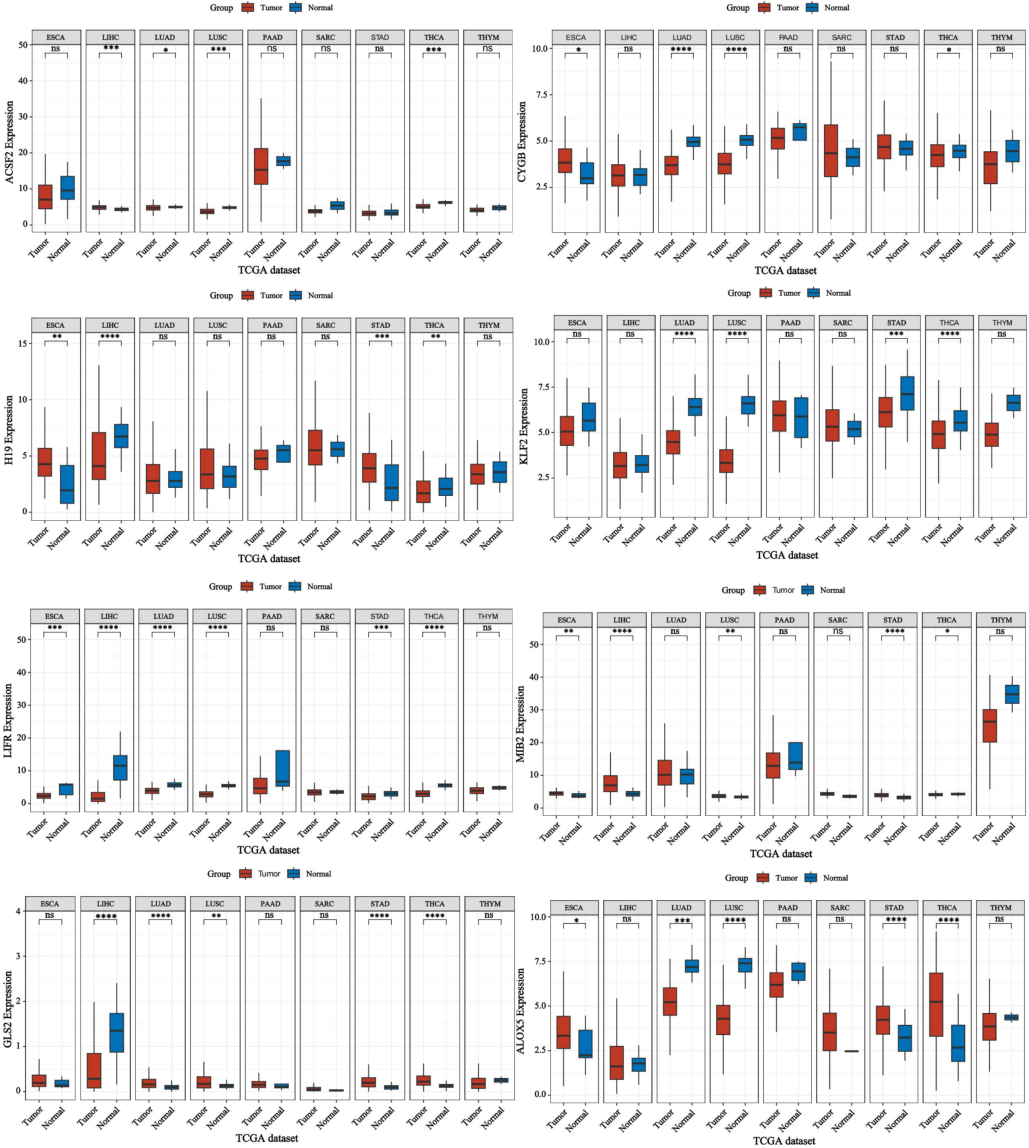
Expression of ferroptosis-related genes in various cancers. Forest plot: This shows the univariate Cox analysis results of a single gene in multiple tumors, including p-value, hazard ratio HR, confidence interval of HR, β value, Wald value, and standard error SE. Asterisks (*) stand for significance levels, where * indicates p < 0.05, ** indicates p < 0.01, *** indicates p < 0.001, and **** indicates p < 0.0001.

Nonetheless, ferroptosis is also critically implicated in other malignancies, including gastric, esophageal, lung, and colorectal cancers (97, 98). Therefore, we also conducted relevant bioinformatics analysis (**Figures 8**). Compared to other cancers, thyroid cancer exhibits unique ferroptosis dynamics. While iron overload and lipid peroxidation are common features of ferroptosis across malignancies, the specific involvement of thyroid-specific molecules like GPX4 and SLC7A11 in regulating ferroptosis pathways in thyroid cancer is more pronounced (43). In advanced thyroid cancer, the balance between iron accumulation, lipid peroxidation, and antioxidant systems such as GPX4 and FTH1 is critical for regulating ferroptosis. In aggressive thyroid cancers like ATC, dysregulation of these components contributes to reduced ferroptosis and increased drug resistance (42). Differentiating between the ferroptotic balance in thyroid cancer and other cancers, such as lung or colorectal cancer, may reveal tumor-specific vulnerabilities that can be targeted therapeutically.

Furthermore, the interplay between ferroptosis and immunotherapy constitutes an innovative approach to cancer treatment. The induction of ferroptosis has the potential to overcome chemoresistance, thereby augmenting therapeutic efficacy. Although the significance of ferroptosis is well-documented in various cancer types, many facets of its underlying mechanisms and regulatory pathways remain insufficiently understood and require comprehensive investigation (80).

For instance, ferroptosis is distinguished by specific cellular and subcellular morphological alterations, as well as unique molecular signatures. Nevertheless, the intersections between ferroptosis and other forms of cell death present considerable challenges (99). A primary research objective is to differentiate ferroptosis from other forms of cell death and to develop specific detection markers and methodologies. An in-depth investigation into the mechanisms and regulatory controls of ferroptosis is essential for its potential application in cancer therapy. Achieving a clearer distinction between ferroptosis and other types of cell death will enhance the evaluation and treatment strategies for cancer (100).

Furthermore, ferroptosis is critically implicated in the initiation and advancement of numerous diseases, including cancer and neurodegenerative disorders. In the context of thyroid cancer, ferroptosis encompasses interactions with coding genes, non-coding genes, environmental factors, and ROS, culminating in the establishment of a complex and interconnected network. Specific coding genes, such as SLC7A11, are instrumental in regulating intracellular iron and lipid peroxide levels, while non-coding genes, such as Circ_0067934, influence the miR-545-3p/SLC7A11 signaling pathway, thereby modulating ferroptosis (101). Environmental factors, including oxidative stress and nutritional status, can induce or inhibit ferroptosis, with ROS driving lipid peroxidation and the production of toxic substances (22, 29). Several compounds have been shown to modulate ferroptosis in thyroid cancer, including sorafenib, which inhibits oxidative stress pathways and induces ferroptosis. Studies have also examined elastin and RSL3, both of which have demonstrated potential to induce ferroptosis in thyroid cancer cell lines, offering new therapeutic avenues. These interconnected factors collectively affect the initiation and progression of thyroid cancer. Additionally, the relationship between ferroptosis and drug resistance, particularly with treatments such as sorafenib, suggests its potential in modulating the drug responsiveness of thyroid cancer. However, further research is needed to fully elucidate how ferroptosis can be harnessed to counteract drug resistance and improve therapeutic outcomes in thyroid cancer.

Recently, there have been some related studies on ferroptosis in the actual clinical application of thyroid cancer. For instance, GPX4 inhibitors, which disrupt the antioxidative defenses of cancer cells, have demonstrated differential ferroptosis effects in thyroid cancer models, providing a promising strategy for overcoming drug resistance (77). Furthermore, curcumin has been shown to activate ferroptosis in follicular thyroid cancer by increasing HO-1 expression, further supporting the therapeutic potential of ferroptosis in thyroid cancer (63). The continued exploration of ferroptosis in thyroid cancer offers hope for developing targeted therapies that could significantly improve treatment outcomes, particularly in cases where conventional approaches have limited effectiveness (43). However, challenges remain in the clinical application of these therapies.

One of the primary obstacles is the lack of specific biomarkers for ferroptosis, which makes it difficult to monitor the effectiveness of treatment in real-time and to identify patients who would benefit most from ferroptosis-inducing therapies (102). Additionally, the tumor microenvironment plays a crucial role in modulating ferroptosis, and its complexity—such as the presence of antioxidants and altered iron metabolism—can hinder therapeutic efficacy (103). Furthermore, ensuring selectivity in targeting cancer cells without inducing toxicity in normal tissues remains a significant challenge (104). The exact mechanism of ferroptosis induction and its interaction with cancer cells is also not fully understood, and further studies are needed to elucidate how this pathway can be efficiently manipulated (43).

To translate these findings into clinical practice, further investigation is needed to elucidate the molecular mechanisms of ferroptosis, explore potential combinatory therapies, and develop robust biomarkers. Such efforts will not only enhance understanding of ferroptosis in thyroid cancer but also pave the way for more effective and targeted treatments. Finally, in the transformation of clinical application, there are still some difficulties such as the side effects of current targeted therapy for advanced thyroid cancer, the possible intolerance of ferroptosis targeting, and the long cycle of clinical research.

In conclusion, despite the need for further investigation to elucidate the specific mechanisms and roles of ferroptosis, this phenomenon has introduced a novel paradigm in disease research. The emerging clinical significance of ferroptosis in the pathogenesis, progression, and treatment of diseases represents an expanding field of study. Existing research indicates that ferroptosis mechanisms significantly contribute to the development of thyroid cancer. This paper provides a comprehensive overview of these mechanisms with the hope that they may offer alternative therapeutic strategies when locally advanced neoadjuvant therapy or conventional treatments for advanced thyroid cancer prove ineffective. Therefore, continued research into ferroptosis in thyroid cancer is essential to improve patient outcomes.

## 7 Conclusions

This review highlights the emerging connection between ferroptosis and thyroid cancer, emphasizing its potential as a therapeutic target. Ferroptosis, through its unique iron-dependent cell death mechanisms, offers a new approach to address treatment-resistant thyroid cancers. Despite current therapies, aggressive malignancies necessitate innovative strategies. However, significant questions remain concerning the molecular interplay between ferroptosis pathways and thyroid cancer progression. The clinical application of ferroptosis as a treatment strategy is nascent, and rigorous clinical trials are needed to establish its efficacy and safety. Future research must focus on elucidating the mechanisms by which ferroptosis impacts thyroid cancer and validating related biomarkers for clinical use. Identifying key regulatory genes and pathways is crucial for developing targeted therapies. Advancements in this field hold promise for developing innovative therapies, improving patient survival, and enhancing quality of life, marking a new era in precision oncology.

## 8 Methods

To investigate ferroptosis-related genes and their potential role in thyroid cancer, we conducted bioinformatics analyses using several publicly available databases. Specifically, we utilized the FerrDb database to identify known ferroptosis-related genes. FerrDb is a comprehensive resource that provides information on ferroptosis-related mechanisms and associated genes. In addition, we used The Cancer Genome Atlas (TCGA) and Gene Expression Omnibus (GEO) datasets to analyze the expression patterns of these genes in thyroid cancer and assess their potential clinical significance. Data analysis was performed using R programming and relevant bioinformatics packages to identify ferroptosis-related genes and their biological roles in thyroid cancer.
